# Interplay between genetically predicted gut microbiota and chronic apical periodontitis: a Mendelian randomization analysis

**DOI:** 10.1080/20002297.2026.2650930

**Published:** 2026-03-31

**Authors:** Lijing Zhu, Yu Xie, Xuechao Yang

**Affiliations:** a Department of Endodontics, School and Hospital of Stomatology, Guangdong Engineering Research Center of Oral Restoration and Reconstruction & Guangzhou Key Laboratory of Basic and Applied Research of Oral Regenerative Medicine, Guangzhou Medical University, Guangzhou, People's Republic of China; b School and Hospital of Stomatology, Guangdong Engineering Research Center of Oral Restoration and Reconstruction & Guangzhou Key Laboratory of Basic and Applied Research of Oral Regenerative Medicine, Guangzhou Medical University, Guangzhou, People's Republic of China

**Keywords:** Gut microbiota, chronic apical periodontitis, Mendelian randomization, causal effect, genome-wide association study

## Abstract

**Background:**

Whether alterations in the gut microbiota play a causal role in chronic apical periodontitis (CAP) remains uncertain. A two-sample Mendelian randomization (MR) analysis was used in this study to assess the potential causal effect of gut microbiota on CAP risk.

**Methods:**

Gut microbiota summary statistics were from the MiBioGen consortium’s largest GWAS, and CAP data from the FinnGen project. The inverse variance–weighted method was the main analytic approach, and sensitivity analyses verified instrument robustness.

**Results:**

Two microbial taxa showed positive causal effect with CAP: *Lachnospiraceae FCS020 group* (odds ratio [OR] = 1.244, 95% confidence interval [CI]: 1.059–1.461, *p* = 0.008) and *Ruminococcus2* (OR = 1.196, 95% CI: 1.039–1.377, *p* = 0.013). Conversely, *Escherichia-Shigella* exhibited an inverse genetic association with CAP risk (OR = 0.746, 95% CI: 0.620–0.897, *p* = 0.002). Sensitivity analyses supported the stability of these findings and confirmed that the effect estimates were directionally consistent, thereby strengthening confidence in the primary results.

**Conclusions:**

This MR analysis provides evidence of a genetic relationship between gut microbiota composition and susceptibility to CAP. The findings broaden our understanding of microbial changes in CAP-affected tissues, and suggest a genetic association between alterations in the composition of the gut microbiota and CAP risk.

## Introduction

Chronic apical periodontitis (CAP) is a persistent inflammatory condition characterised by progressive destruction of periradicular tissues and alveolar bone resorption [1]. Due to its high prevalence and long-term complications, CAP has emerged as a significant public health concern in oral medicine [2].

While previous investigations of the pathogenesis of oral disorders have typically focused on the involvement of oral microbiota [3], recent studies have highlighted the importance of the ‘oral–gut axis’, a paradigm that emphasises the complex crosstalk between the oral and gut microbiota [4]. As the body's largest microbial ecosystem, the gut microbiome exerts far-reaching effects on metabolic, immune and inflammatory pathways [5,6]. Therefore, interest has grown in exploring whether disturbances of the gut microbiota may also contribute to inflammatory oral lesions. Various studies have reported causal associations between the gut microbiota and oral diseases, such as gingivitis, periodontitis and oral ulcers [7–9]. Among these oral disorders, CAP is a common secondary development of pulpitis-associated infection [10]. Importantly, periodontitis, pulpitis and periapical periodontitis can coexist, accompanied by reciprocal exacerbation of their infectious cascades, suggesting the potential involvement of similar microbial pathogens. However, it is not clear whether there is a causal relationship between the intestinal microbiota and CAP. Clarifying this link is essential for advancing the present understanding of the mechanistic basis for CAP pathogenesis.

Mendelian randomisation (MR), based on the principle that genotypes are randomly assigned at gamete formation, is frequently used to explore causal pathways [11]. MR uses inherited genetic variation, most often in the form of single nucleotide polymorphisms, as instrumental variables to infer causality while minimising confounding and reverse causation [12]. This approach has been widely applied to clarify risk factors for diverse outcomes, including cancer [13] and cardiovascular diseases [14,15].

Here, we conducted a two-sample MR analysis integrating large-scale GWAS data pertaining to the gut microbiota and CAP. By evaluating 192 gut microbial taxa, we aimed to identify taxa exerting causal effects on CAP risk, to provide new insights into the microbial underpinnings of this disease, and to establish a theoretical basis for further mechanistic and clinical investigations of this condition.

## Methods

### Study design

A two-sample MR framework was employed to evaluate the potential causal effect of gut microbiota on CAP risk. In the present study, gut microbiota taxa were defined as the exposure variable, and CAP as the outcome variable. This design rests on three core assumptions: (1) the genetic instruments (instrumental variables, IVs) must be robustly associated with characteristics of the gut microbiota; (2) these instruments should be independent of confounding factors; and (3) these IVs must influence CAP solely through their effects on the gut microbiota rather than via alternative pathways. The genome-wide association studies (GWAS) datasets used in this study had been previously approved by their respective ethics committees, and all participants had provided informed consent.

### Gut microbiota data source

MiBioGen, one of the largest databases of GWAS of microbiota, offers compelling advantages for analysis of the gut microbiota [16]. The database includes 14,306 samples, and integrates 16S ribosomal RNA gene sequencing data with genotyping information from 18,340 multi-ethnic participants across 24 cohorts spanning 11 countries (the USA, Canada, Israel, South Korea, Germany, Denmark, the Netherlands, Belgium, Sweden, Finland and the UK). The extensive sample size can effectively minimise research bias, ensuring high reliability and generalisability of the analytical outcomes [16]. This resource includes summary statistics for 192 bacterial taxa spanning multiple taxonomic levels: 116 genera, 31 families, 20 orders, 16 classes and 9 phyla (Supplementary Table 1).

### CAP data source

Outcome data were obtained from the R10 release of the FinnGen project [17]. The diagnosis of CAP was based on the K04.5 classification within the ICD-10 codes. This dataset includes 7,101 CAP cases and 272,252 controls of European ancestry. The controls were recruited from the general population, excluding those diagnosed with diseases involving the oral cavity, salivary glands, or jaws. Comprehensive information on the study design, participant selection criteria and methodological procedures is publicly accessible via the FinnGen database (https://www.finngen.fi/).

### Instrumental variable selection

We applied the ‘TwoSampleMR’ package in R (v 4.3.1) to perform MR analyses exploring the causal relationship between the composition of the gut microbiota and CAP. To satisfy MR assumptions, the following criteria were used to select SNPs as instrumental variables: (1) SNPs had to meet the genome-wide significance threshold (*p* < 1 × 10^−5^) for association with gut microbiota traits [18], with all candidate SNPs being listed in Supplementary Table 2; (2) reference panels constructed from European samples in the 1000 Genomes Project were used to assess linkage disequilibrium among the SNPs. SNPs in linkage disequilibrium were pruned at R^2^ < 0.001 within a 10,000-kb window; (3) R^2^ and F statistics were calculated to avoid weak instrument bias; SNPs with F < 10 or a minor allele frequency ≤ 0.01 were excluded; and (4) palindromic SNPs were removed to prevent strand ambiguity.

### Statistical and power analyses

We applied four complementary MR approaches to evaluate potential causal effects, with the random-effects inverse variance-weighted (IVW) model designated as the primary analytic method [19]. MR-Egger regression, the weighted median estimator and the weighted mode method served as secondary approaches to corroborate the findings. To assess potential heterogeneity among the selected SNPs, we calculated Cochran's Q statistics, while directional pleiotropy was evaluated using the MR-Egger intercept test and the MR-PRESSO global test. We also conducted leave-one-out analyses to determine whether any single SNP exerted a disproportionate influence on the overall estimates. Finally, because this MR study aimed to identify novel microbial targets across 192 gut taxa for the prevention or treatment of CAP, we performed MR Steiger filtering to establish the likely direction of causality between each microbial trait and CAP [20]. A ‘TRUE’ MR Steiger outcome indicated an effect proceeding from gut microbiota to CAP (*p* < 0.05), whereas a ‘FALSE’ result implied reverse causation. Power analysis was performed for the two-sample MR estimates using sample sizes of 18,340 for exposure and 279,353 for outcome at a significance level of 0.05.

## Results

### Instrumental variable selection for gut microbiota taxa

**Figure 1.**
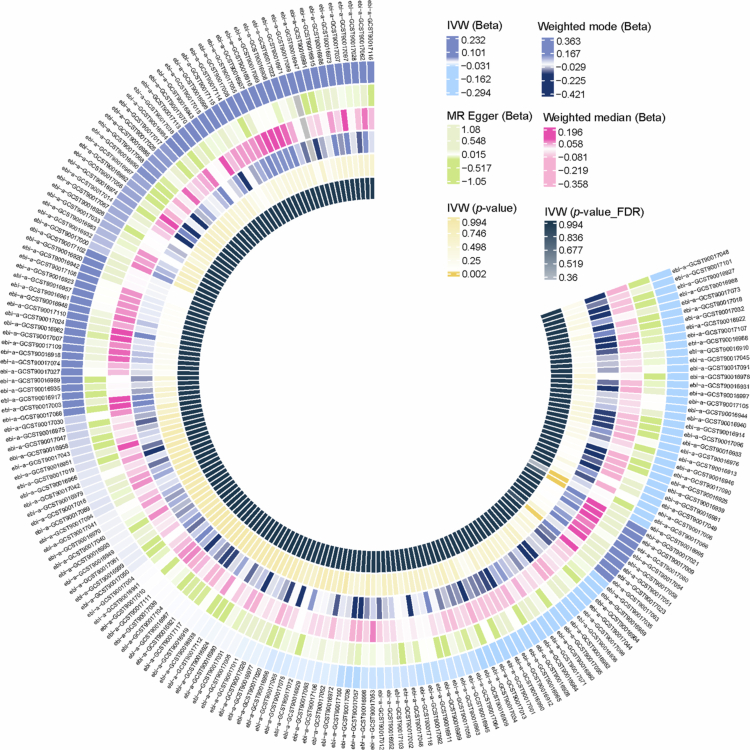
The causal association between the gut microbiota and chronic apical periodontitis. From the inner circle to the outer circle, the following statistical methods are presented: weighted mode, weighted median, MR-Egger and inverse variance-weighted.

Figure 1 provides a summary of the associations between genetically predicted gut microbial taxa and CAP risk. Across all analyses, we identified 2,094 independent SNPs meeting the genome-wide significance threshold as instrumental variables for gut microbiota traits. A threshold value of F < 10 was used to define ‘weak IV’. In this study, all instruments displayed F-statistics exceeding 10, with a mean F-statistic of 22.65, thereby minimising the likelihood of weak instrument bias. Detailed SNP-level information is provided in Supplementary Table 2.

### Causal relationship between gut bacterial taxa and chronic apical periodontitis

**Figure 2.**
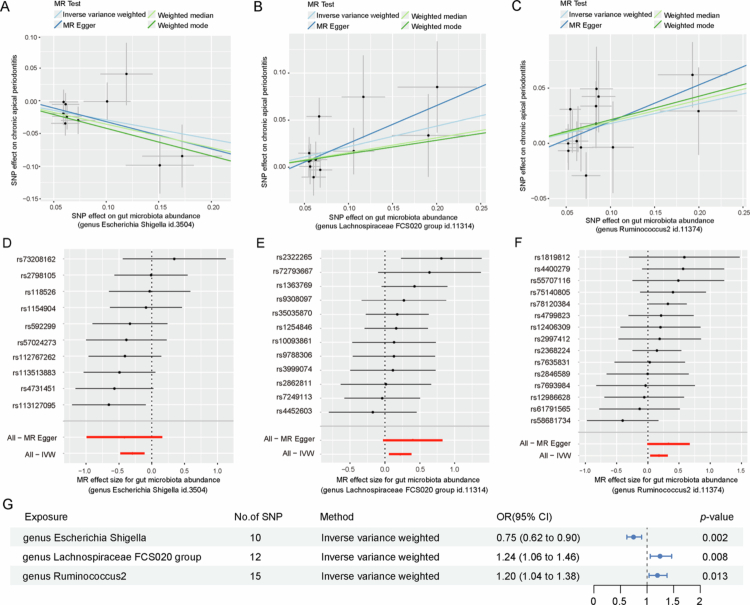
Scatter plots and forest plots corresponding to the causal relationship between the gut microbiota and chronic apical periodontitis. Scatter plots: (A) *Escherichia-Shigella*, (B) *Lachnospiraceae FCS020 group* and (C) *Ruminococcus2*; Forest plots: (D) *Escherichia-Shigella*, (E) *Lachnospiraceae FCS020 group* and (F) *Ruminococcus2*.

As shown in Table 1 and Figure 2, three bacterial taxa were significantly linked to CAP causally. Two taxa exhibited positive associations: *Lachnospiraceae FCS020 group* (OR = 1.244, 95% CI: 1.059–1.461, *p* = 0.008) and *Ruminococcus2* (OR = 1.196, 95% CI: 1.039–1.377, *p* = 0.013). The results of the IVW analysis showed that *Lachnospiraceae FCS020 group* and *Ruminococcus2* were potentially linked to a higher risk of CAP. Results from the supplementary MR methods were directionally consistent with the IVW estimates (Figure 2).

**Table 1.** MR estimates for the association between gut microbiota and chronic apical periodontitis.

Taxa	MR method	SNPs	Beta	SE	OR (95%CI)	*p*-value
Genus: *Escherichia-Shigella* (id.3504)	Inverse variance weighted	10	−0.293	0.094	0.746 (0.620–0.897)	0.002
	MR Egger	10	−0.416	0.293	0.659 (0.372–0.171)	0.193
	Weighted median	10	−0.358	0.134	0.699 (0.538–0.909)	0.007
	Weighted mode	10	−0.421	0.207	0.656 (0.437–0.985)	0.073
Genus: *Lachnospiraceae FCS020 group* (id.11314)	Inverse variance weighted	12	0.218	0.082	1.244 (1.059–1.461)	0.008
	MR Egger	12	0.399	0.217	1.492 (0.974–2.284)	0.095
	Weighted median	12	0.156	0.109	1.169 (0.944–1.448)	0.153
	Weighted mode	12	0.143	0.159	1.154 (0.845–1.577)	0.388
Genus: *Ruminococcus2* (id.11374)	Inverse variance weighted	15	0.179	0.072	1.196 (1.039–1.377)	0.013
	MR Egger	15	0.331	0.174	1.392 (0.990–1.956)	0.079
	Weighted median	15	0.196	0.094	1.217 (1.011–1.464)	0.038
	Weighted mode	15	0.213	0.150	1.237 (0.921–1.661)	0.179

The *Escherichia-Shigella* (OR = 0.746, 95% CI: 0.620–0.897, *p* = 0.002) was inversely associated with CAP (Table 1 and Figure 2), suggesting that *Escherichia-Shigella* in the human gut is causally related to a lower risk of CAP. Estimates from all MR approaches of all bacterial taxa are summarised in Supplementary Table 3. Directionality testing using MR-Steiger filtering further evaluated the putative causal flow between gut microbiota and CAP (Supplementary Table 4). The three bacterial taxa, *Lachnospiraceae FCS020 group*, *Ruminococcus2* and *Escherichia-Shigella* identified in this study all passed the MR-Steiger filtering.

### Sensitivity and power analyses

**Figure 3.**
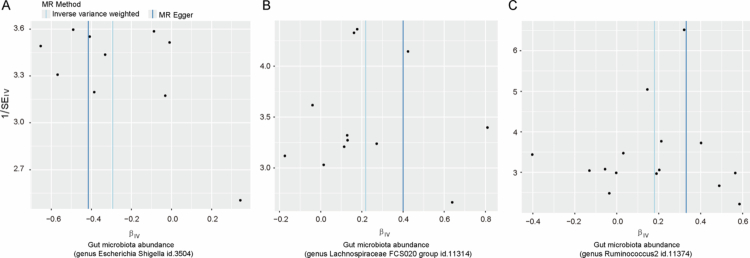
Funnel plots corresponding to the causal relationship between the gut microbiota and chronic apical periodontitis. (A) *Escherichia-Shigella*, (B) *Lachnospiraceae FCS020 group* and (C) *Ruminococcus2.*

**Figure 4.**
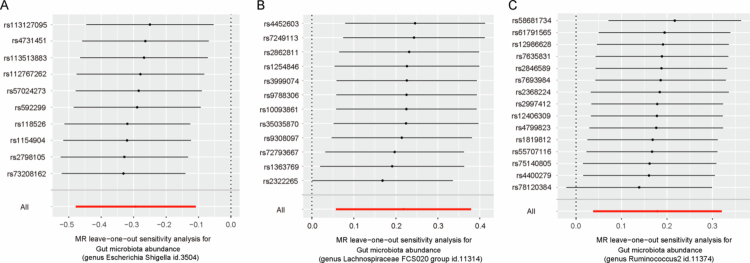
Leave-one-out analysis of the impact of the gut microbiota on chronic apical periodontitis risk. (A) *Escherichia-Shigella*, (B) *Lachnospiraceae FCS020 group* and (C) *Ruminococcus2.*

Sensitivity tests demonstrated no evidence of substantial heterogeneity or directional pleiotropy for the three taxa associated with CAP causally. As assessed by Cochran's Q test, no significant heterogeneity was detected in three bacterial taxa (all *p* > 0.05; Table 2 and Supplementary Table 5). In addition, no horizontal pleiotropy of instrumental variables was identified, as supported by the MR-Egger intercept test (all *p* > 0.05; Table 2 and Supplementary Table 6) and the MR-PRESSO global test (*p* > 0.05; Table 2). Funnel plots showed symmetrical distributions of SNP effect estimates, suggesting a low likelihood of bias (Figure 3). Additionally, leave-one-out analysis also revealed that no single SNP disproportionately influenced the associations across the three identified taxa, confirming the robustness of the results (Figure 4).

**Table 2.** Sensitivity analysis of the results of MR analysis of gut microbiota and chronic apical periodontitis.

Exposure	IVW (heterogeneity)		MR- Egger (heterogeneity)		MR-Egger (pleiotropy)		MR-PRESSO (pleiotropy)	
	*p*-value	Q	*p*-value	Q	*p*-value	intercept	*p*-value	RSSobs
Genus: *Escherichia-Shigella* (id.3504)	0.539	7.949	0.458	7.754	0.671	0.009	0.580	9.616
Genus: *Lachnospiraceae FCS020 group* (id.11314)	0.607	9.166	0.594	8.352	0.388	−0.015	0.650	10.734
Genus: *Ruminococcus2* (id.11374)	0.712	10.663	0.714	9.743	0.355	−0.013	0.738	12.340

A power analysis verified that adequate statistical power was achieved to detect the observed causal effects in this study, given exposure and outcome sample sizes of 18,340 and 279,353, respectively.

## Discussion

This MR study identified a genetic relationship between three bacterial taxa, *Lachnospiraceae FCS020 group*, *Ruminococcus2* and *Escherichia-Shigella* and CAP. The study findings showed inconsistencies among the IVW, MR-Egger and weighted median models. In terms of model selection, MR models balance power and robustness, IVW is optimal for valid instruments and MR-Egger and weighted median approaches mitigate the risk of pleiotropy. The strategy used ensured the reliability of the causal inference by the integration of pleiotropy tests with model characteristics, which is consistent with current MR guidelines [21]. No significant pleiotropy was detected in the analysis of genetic variants and CAP (Egger intercept *p* > 0.05; MR-PRESSO global test *p* > 0.05), indicating the reliability of the IVW results.

Studies have shown that gut microorganisms and their metabolites, such as lipopolysaccharides, can be transported through the bloodstream to the alveolar bone, leading to inflammation of distal organs [22]. One of the clinical symptoms of CAP is bone destruction, in which short-chain fatty acids (SCFAs) produced by intestinal microorganisms can regulate the balance between osteoclasts and osteoblasts [23,24] thereby potentially playing a role in this process. Our MR analysis identified two taxa as being causally associated with CAP risk, including *Lachnospiraceae FCS020 group* and *Ruminococcus2*. The *Lachnospiraceae FCS020 group*, a genus within the *Lachnospiraceae* family, is known for producing SCFAs. Butyrate, one of the key SCFAs produced by this group, influences intestinal physiology and immune regulation by promoting regulatory T cell differentiation through histone acetylation. Perturbations in gut microbial composition and metabolite production can alter immune cell activation and cytokine secretion [25]. Notably, in the context of chronic inflammation induced by a high-fat diet, an increased abundance of *Lachnospiraceae FCS020 group* was associated with heightened IL-2RA concentrations, reflecting a pro-inflammatory state [26]. Given that CAP is a chronic immune-inflammatory disease originating from pulpal infection and extending into periradicular tissues, it is plausible that *Lachnospiraceae FCS020 group* contributes to disease pathogenesis by modulating systemic immune or inflammatory pathways.

*Ruminococcus2*, another SCFA-producing genus, also emerged as a potential genetic risk factor. This organism has been implicated in thyroid cancer [27,28] and endocarditis [29], although in breast cancer a higher abundance appears protective (OR = 0.77, *p* = 4.91 × 10^−4^, FDR = 0.04) [30]. Moreover, *Ruminococcus2* has been linked to attenuation of intestinal inflammation in ulcerative colitis via CCL4-mediated mechanisms [31]. Other members of this genus, such as *Ruminococcus gnavus*, have been shown to provoke Th2-driven inflammation and eosinophilia, with eosinophils potentially disseminating through the circulation to other inflamed tissues [32]. These observations suggest that systemic recruitment and migration of inflammatory cells may underlie how certain gut bacteria influence CAP risk. Further mechanistic studies, including those utilising microbiome profiling and experimental models, are needed to elucidate these pathways.

In contrast, our MR analysis revealed a negative causal association between CAP and *Escherichia-Shigella*, a Gram-negative enterobacterium classically recognised as a pathogen causing shigellosis [33]. Elevated levels of *Escherichia-Shigella*have been documented in multiple disorders, including cancer [34,35], inflammatory bowel disease [36] and Kawasaki disease [37]. In the context of oral conditions, no genetic causal effects have been reported for periodontitis [8], oral ulcers [9] and gingivitis [7], although increased abundance has been observed in oral lichen planus lesions [38]. Despite its reputation as a pro-inflammatory taxon [39] associated with heightened circulating antibody levels [40], our findings indicate a potentially protective effect of *Escherichia-Shigella* against CAP. These unexpected results warrant additional studies to clarify the underlying biological mechanisms and explore whether this taxon exerts direct or indirect modulation of oral immune responses.

Organ dysfunction, including heart failure [41] and diabetes [42], can modulate the composition of the gut microbiota. A bidirectional relationship between diabetes and periodontitis has been documented [43,44], associated with the systemic dissemination of gut pathogens and their metabolites via the circulatory system [22]. Alterations in the gut microbiota composition may thus contribute to the mutual aggravation of these two conditions. Notably, prior studies have revealed partial overlap between the microbiomes of the gut and the pancreas, albeit with varying degrees of similarity [45]. Given the complexity of microbial distributions in the systems of the human body, future research should verify these findings through sequencing of larger clinical cohorts, as well as animal experiments. Nevertheless, the present study offers preliminary theoretical support for subsequent identification of target microbial taxa associated with CAP lesions.

Previous studies have established a causal link between gut microbiota and the risk of gingivitis [7] and periodontitis [8], with correlations also observed for periodontitis, pulpitis and CAP. However, the present findings reveal that CAP-associated microbial changes do not overlap with those found for other oral diseases, indicating differences in the dominant bacterial pathogens driving these disorders.

This study possesses several strengths. First, the MR framework mitigates confounding and reverse causation, thereby enhancing the credibility of causal inferences. Second, we leveraged large-scale genetic datasets, enabling more robust statistical power compared to traditional observational designs. Third, multiple sensitivity analyses including MR-Egger regression, Cochran's Q testing, MR-PRESSO testing and MR Steiger filtering were performed to assess horizontal pleiotropy, heterogeneity and directionality of effect, reinforcing the stability of our conclusions. Finally, using an MR approach allowed us to evaluate a broad spectrum of gut taxa simultaneously, identifying previously unrecognised microbial correlates of CAP.

Nonetheless, several limitations should be acknowledged. First, there are differences in terms of datasets, and the reliance of the MiBioGen data on 16S rRNA sequencing, which has lower resolution than whole-genome sequencing, may have amplified this bias. Future studies using quantification of absolute abundance and higher-resolution methods (e.g. whole-genome metagenomic sequencing) will provide greater clarification of the microbial causal relationships. Given the low trait heritability of the microbiome and the above limitations, the findings of the present study should be considered exploratory. Second, the present analyses were restricted to individuals of European ancestry, thereby potentially limiting the generalisability of the findings to other populations, particularly Asian cohorts such as Chinese patients. Prospective studies and replication in ethnically diverse populations are needed to validate these associations and to clarify their translational relevance. Topics for future research may include the collection of CAP-associated apical tissues (e.g. granulomas, cysts) and normal controls (e.g. orthodontically extracted healthy teeth or non-inflamed prophylactically removed wisdom teeth), followed by microbiome sequencing to explore microbial abnormalities in CAP samples.

In summary, this two-sample MR study provides genetic evidence that the gut microbiota composition may influence CAP risk in European populations. Specifically, *Lachnospiraceae FCS020 group* and *Ruminococcus2* were positively associated with CAP, whereas *Escherichia-Shigella* showed a negative association with this condition. The findings of this study provide a preliminary theoretical foundation for further analyses of microbial changes in CAP-associated tissues, leading to the identification of specific factors linked to the risk of CAP.

## Supplementary Material

Supplementary Tables.xlsx Supplementary Tables.xlsx
